# Biodegradation of lignin and nicotine with white rot fungi for the delignification and detoxification of tobacco stalk

**DOI:** 10.1186/s12896-016-0311-8

**Published:** 2016-11-21

**Authors:** Yulong Su, He Xian, Sujuan Shi, Chengsheng Zhang, S. M. Nuruzzaman Manik, Jingjing Mao, Ge Zhang, Weihong Liao, Qian Wang, Haobao Liu

**Affiliations:** 1 Key Laboratory of Tobacco Biology and Processing, Tobacco Research Institute, Chinese Academy of Agricultural Sciences, Qingdao, 266101 People’s Republic of China; 2 Qingdao No.9 High School, Qingdao, 266012 Shandong Province China; 3 College of Agriculture and Plant Protection, Qingdao Agricultural University, Qingdao, 266109 China; 4 Shandong Lukang Drugs Group, Jining, 272001 China

**Keywords:** Tobacco stalk, Nicotine degradation, Delignification, *Phanerochaete chrysosporium*, Lignocellulolytic enzymes

## Abstract

**Background:**

Tobacco stalk is one kind of abundant crop residues in China. The high lignification of tobacco stalk increases its reusing cost and the existing of nicotine will cause serious pollution. The biodegradation of lignocellulosic biomass has been demonstrated to be an environmental and economical approach for the utilization of plant stalk. Meanwhile, many nicotine-degrading microorganisms were found in nature. However, microorganisms which could degraded both nicotine and lignin haven’t been reported. Therefore, it’s imperative to find some suitable microorganisms to break down lignin and simultaneously remove nicotine in tobacco stalk.

**Results:**

The nicotine in tobacco stalk could be degraded effectively by *Trametes versicolor*, *Trametes hirsute* and *Phanerochaete chrysosporium*. The nicotine content in tobacco stalk was lowered to below 500 mg/kg (a safe concentration to environment) after 10 days of fermentation with *Phanerochaete chrysosporium* and *Trametes versicolor*, and 15 days with *Trametes hirsute*. The degradation rate of lignin in the fermented tobacco stalk was 37.70, 51.56 and 53.75% with *Trametes versicolor*, *Trametes hirsute* and *Phanerochaete chrysosporium*, respectively. Meanwhile, 24.28% hemicellulose was degraded by *Phanerochaete chrysosporium* and 28.19% cellulose was removed by *Trametes hirsute*. Through the enzyme activity analysis, the main and highest ligninolytic enzymes produced by *Phanerochaete chrysosporium*, *Trametes hirsute* and *Trametes versicolor* were lignin peroxidase (88.62 U · L^−1^), manganese peroxidase (100.95 U · L^−1^) and laccase (745.65 U · L^−1^). Meanwhile, relatively high and stable cellulase activity was also detected during the fermentation with *Phanerochaete chrysosporium*, and the highest endoglucanase, exoglucanase and filter paper enzyme activities were 0.38 U · mL^−1^, 0.45 U · mL^−1^ and 0.35U · mL^−1^, respectively. Moreover, the products in the fermentation of tobacco stalk with *P. chrysosporium* were identified with GC-MS, besides the chemicals produced in the degradation of lignin and nicotine, some small molecular valuable chemicals and fatty acid were also detected.

**Conclusions:**

Our study developed a new method for the degradation and detoxification of tobacco stalk by fermentation with white rot fungi *Phanerochaete chrysosporium* and *Trametes hirsute*. The different oxidative enzymes and chemical products detected during the degradation indicated a possible pathway for the utilization of tobacco stalk.

**Electronic supplementary material:**

The online version of this article (doi:10.1186/s12896-016-0311-8) contains supplementary material, which is available to authorized users.

## Background

Tobacco is an important cash crop in China and has been planted in many farming areas. It has been estimated that about 3.2 million tons of tobacco stalk were produced annually in China [1]. The reusing of tobacco stalk is costly due to its high lignification. Most of tobacco stalk is discarded in the field directly and the nicotine in tobacco stalk can easily permeate into the soil, affecting its ecological structure and also polluting the ground water. It is reported that the average nicotine content in tobacco stalk is up to 3800 mg/kg [2] and the nicotine-containing waste would be classified as “toxic and hazardous”, when the concentration of nicotine exceeds 500 mg/kg dry weight [3]. Therefore, it is imperative to remove nicotine from tobacco stalk so as to make better utilization of tobacco stalk.

Significant progresses have been made in the utilization of tobacco stalk such as making fiberboard, tobacco sheet, acticarbon or extracting chemicals [4–7]. However, all of these methods were deficient due to the terrible pollution on the environment or the high cost, and it is necessary to explore new approaches to the safe and sustainable utilization of tobacco stalk. The biodegradation of lignocellulosic biomass has been demonstrated to be an environmental-friendly and economical way for the reusing of plant stalk [8]. It is already shown that fungi belonged to *Moniliales Gliocephalias* sp. [9] and *Aspergillus* sp. [10] could degrade lignin in tobacco stalk. However, the microbes that could be used for the biodegradation of tobacco stalk were still limited due to the high lignification and the existing of nicotine. Though many microorganisms have been demonstrated to be able to degradate lignin and nicotine separately [11, 12], microorganisms which could degradate the both haven’t been reported.

This study aimed to screen anti-nicotine microorganisms for the degradation of tobacco stalk, and explore their degradation characteristics. The research will provide a better way for the innocent treatment of tobacco stalk.

## Results and discussion

### Effect of fungi fermentation on nicotine degradation in tobacco stalk

**Fig. 1:**
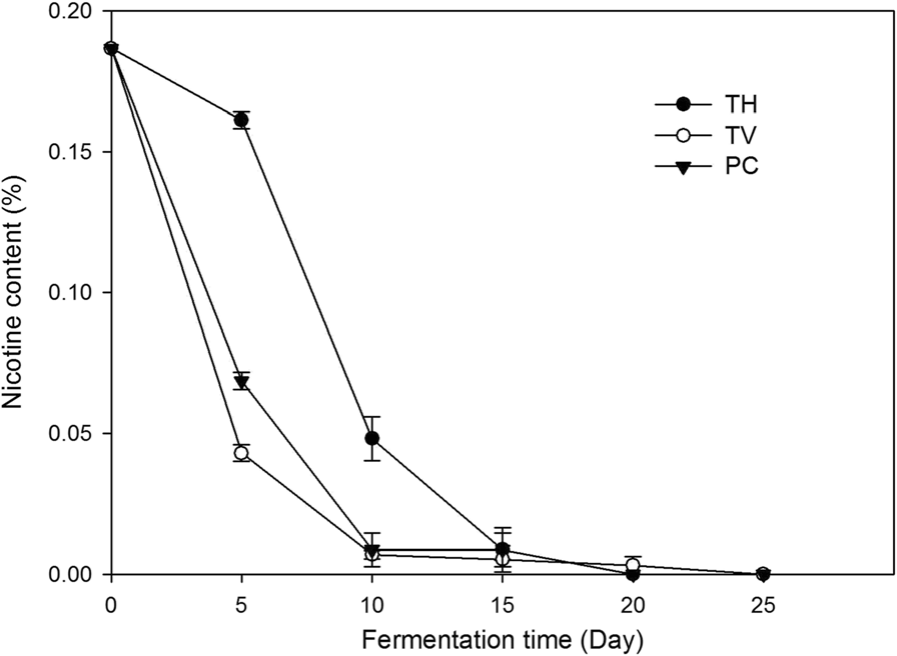
The content of nicotine in tobacco stalk when fermentation with *Phanerochaete chrysosporium*, *Trametes versicolor* and *Trametes hirsute* in solid state. The culture temperature was 28 °C and the relative humidity was 80%, the nicotine content was detected every 5 days until 25 days after inoculation

The concentration of nicotine in tobacco stalk was 1900 mg/kg in this study (Fig. 1). Nicotine has been defined as one kind of the toxic and hazardous chemicals in tobacco wastes [13]. It can contaminate ground water when the concentration exceeds 500 mg/kg in dry weight. Therefore, it is necessary to prevent the contamination of nicotine during the biodegradation of tobacco stalk.

Nicotine concentration analysis showed that all the fungi selected could efficiently degradate nicotine (Fig. 1). For *P. chrysosporium* and *T.versicolor*, the concentration of nicotine in tobacco stalk remained lower than 500 mg/kg after 10 days of fermentation. For *T. hirsute*, it is found that nicotine degradation was accelerated from day 10–15 after inoculation. The nicotine content was less than 550 mg/kg after 15 days fermentation. No residual nicotine was detected in fermented aqueous extracts after 25 days of incubation in all the fungi culture. To our best knowledge, this is the first study showing that nicotine can be degraded by *P. chrysosporium*, *T. versicolor* and *T. hirsute* and making the bio-degradation of nicotine. Lac can oxidize phenolic compounds directly, while all the three enzymes can oxidize divers non-phenolic aromatic compounds with the help of mediators [14], which would be helpful to the degradation of nicotine.

### Effect of fungi fermentation on lignocelluloses degradation in tobacco stalk

It is shown that in the nicotine degradation process, at least 10–15 days of fermentation is necessary to ensure the degradation of nicotine in tobacco stalk to a safe concentration. The lignocelluloses were also monitored during the process to verify the selective degradation of lignin in tobacco stalk with three white rot fungi selected.

The untreated tobacco stalk used in this study was composed of 40.28% hemicellulose, 30.22% cellulose, 21.06% lignin and others. The lignocelluloses biodegradation in tobacco stalk caused by three white rot fungi (*P. chrysosporium*, *T. hirsute* and *T. versicolor*) are listed in Table 1. The result showed that the lignocelluloses decreased gradually with increasing fermentation time. A significantly decrease of hemicellulose was observed in *P. chrysosporium* (24.28%), and cellulose was degraded by 28.19% after 15 days fermentation with *T. hirsute*. Almost the same amount of lignin was degraded by three white rot fungi from 9–15 days, the maximum lignin reduction 53.75 and 51.56% were observed in *P. chrysosporium* and *T. hirsute*, respectively, but only 37.70% lignin in tobacco stalk was removed by *T. versicolor*.

**Table 1:** Changes in lignocelluloses content of tobacco stalk treated with white rot fungi strains during solid state fermentation (dry matter basis)

Strains	Fermentation period (days)	Cellulose content (%)	Cellulose degradation (%)	Hemicellulose content (%)	Hemicellulose degradation (%)	Lignin content (%)	Lignin degradation (%)
**Control** ^**a**^		**30.22**		**40.28**		**21.06**	
*P.chrysosporium*	9	26.96	10.79	37.32	7.35	11.92	43.40
	11	26.22	13.24	35.6	11.62	11.74	44.25
	13	25.96	14.10	33.28	17.38	11.68	44.54
	15	22.32	22.17	30.5	24.28	10.94	53.75
*T. hirsute*	9	28.18	6.75	37.28	7.45	12.96	38.46
	11	24.22	19.85	35.54	11.77	12.5	40.65
	13	23.64	21.77	35.06	12.96	11.88	43.59
	15	21.7	28.19	34.18	15.14	10.2	51.57
*T. versicolor*	9	28.88	4.43	38.46	4.52	15.88	24.60
	11	28.78	4.77	37.36	7.25	15.74	25.26
	13	27.08	10.39	36.36	9.73	15.26	27.54
	15	26.24	13.17	36.02	10.58	13.12	37.70

control^a^: The data in bold was the control, the initial contents of cellulose, hemicellulose and lignin

In our study, hemicellulose was significantly degraded by *P. chrysosporium* and cellulose was significantly degraded by *T. hirsute*, and both of the two fungi showed relative higher lignin degradation rate. This is in accordance with previous reports that selective white-rot fungi utilized hemicellulose and/or cellulose to support their growth [15]. For lignin degradation of tobacco stalk, a maximum of 37.60% lignin degradation rate was reached on day 7 by *Aspergillus* sp. [10] and 39.39% on day 30 by *Moniliales Gliocephalias* sp. [9], meanwhile the enzyme activities were not investigated. The steam explosion pretreatment of the tobacco stalk may be benefit to get a relatively higher lignin degradation rate [16].

The selective degradation of lignin in tobacco stalk is important for the reusing of cellulose in tobacco stalk. The selectivity was evaluated by comparing the relative degradation rate for lignin and cellulose. The percentages of lignin degradation obtained in this study were higher than cellulose, which indicates the three fungal strains could be used in the delignification of tobacco stalk.

### Water-soluble and ester-soluble chemicals after fermentation with *P. chrysosporium*

With the development of synthetic biology, more and more valuable chemical products were found and qualified. Tobacco stalk is rich in lignin and nicotine and identification of the fermented products and the intermediates may be helpful for finding valuable biobased chemicals. In this study, *P. chrysosporium* was shown to degrade the tobacco stalk efficiently. Moreover, it could secrete various enzymes. Therefore, the products in the degradation of tobacco stalk with *P. chrysosporium* were qualified. The total ion chromatographs corresponding to the compounds extracted with ethyl acetate and water from the supernatants of the fermentation stalk by *P. chrysosporium* were analyzed.

According to the degradation pathway of the nicotine and lignin [17, 18], some intermediates during the degradation of nicotine such as myosmine, cotinine, N-methyl nicotinamide, N-methylpyrrolidone, pyridine, 2,3′-dipyridyl, nicotinyl alcohol and niacin were detected in the culture of *P. chrysosporium* (Table 2). Meanwhile, some products from the degradation of lignin were also detected in the culture of *P. chrysosporium* such as 2-furancarboxaldehyde, 5-methyl, 2-furancarboxaldehyde, 5-(hydroxymethyl), vanillin, benzenemethanol, 3, 4-dimethoxy (Table 2). The detection of these chemical products further proves the nicotine-degrading ability of *P. chrysosporium*. Besides the chemicals produced in the degradation of lignin and nicotine, some small molecular valuable chemicals and fatty acid were also detected in cultures of *P. chrysosporium*. Acetoin and acrylic acid were measured in water extractions. Ethyl propionate and propyl acetate were detected in ethyl acetate extractions. Palmitic acid was found in both of them.

**Table 2:** The main components in the cultures of *P. chrysosporium* (80% W) grew on tobacco stalk during solid-state fermentation

The classification of the fermentation products	Retain time (min)	Solvent	Name
Nicotine degradation products	6.99	aqueous phase	Myosmine
	17.63	aqueous phase	Cotinine
	17.75	aqueous and ethyl acetate phase	Nicotinyl alcohol
	19.51	aqueous phase	3-Hydroxypyridine
	22.01	aqueous phase	Nicotinic acid
	23.62	aqueous phase	Pyridine
Lignin degradation products	20.19	aqueous phase	5-Hydroxymethylfurfural
	21.20	aqueous phase	Veratryl alcohol
	20.75	ethyl acetate phase	Vanillin
Small and high molecular organic	3.03	ethyl acetate phase	Ethyl propionate
	3.15	ethyl acetate phase	Propyl acetate
	7.91	aqueous phase	Acetoin
	11.71	aqueous phase	Acrylic acid
	23.98	aqueous phase and ethyl acetate phase	Palmitic acid

The detection of lignin and nicotine degradation intermediates further proved the lignin and nicotine degradation ability of *P. chrysosporium*. It has been demonstrated by Li [19] and Meng [11] that demethylation pathway is the main nicotine degradation pathway performed by fungi. In our study, some intermediates of demethylation pathway were detected in the culture of *P. chrysosporium*, such as cotinine, N-methylmyosmine and pseudooxynicotine. In addition, some different intermediates such as 3-Pyridinol, nicotinyl alcohol and niacin which may be the degradation intermediates were also detected which hint a new nicotine degradation pathway may be present in *P. chrysosporium*. Moreover, the detection of small molecular chemicals and fatty acid provides a possible pathway for production of biobased chemicals by fermentation with tobacco stalk. The degradation pathway of nicotine and the concentration of the products in the degradation of tobacco stalk with *P. chrysosporium* need further research.

### Enzyme activities produced during solid state fermentation of tobacco stalk

Enzyme activities are important characteristics for the degradation of biomass, moreover, the produced enzymes can also be used in various applications. In this study, the detection of enzyme activities were carried out for 25 days to identify the highest enzyme activities. Ligninolytic and cellulolytic enzymes activities were monitored in the degradation process to estimate their potential application in lignocellulolytic enzymes production.

#### Ligninolytic enzyme during solid state fermentation of tobacco stalk

Lac, LiP and MnP are three kinds of common lignin peroxides enzymes secreted in the degradation of lignocelluloses biomass. It was found that different ligninolytic enzymes were secreted by different fungi when fermentation with tobacco stalk. However, not all of the ligninolytic enzymes were produced by a specific fungus.

**Fig. 2:**
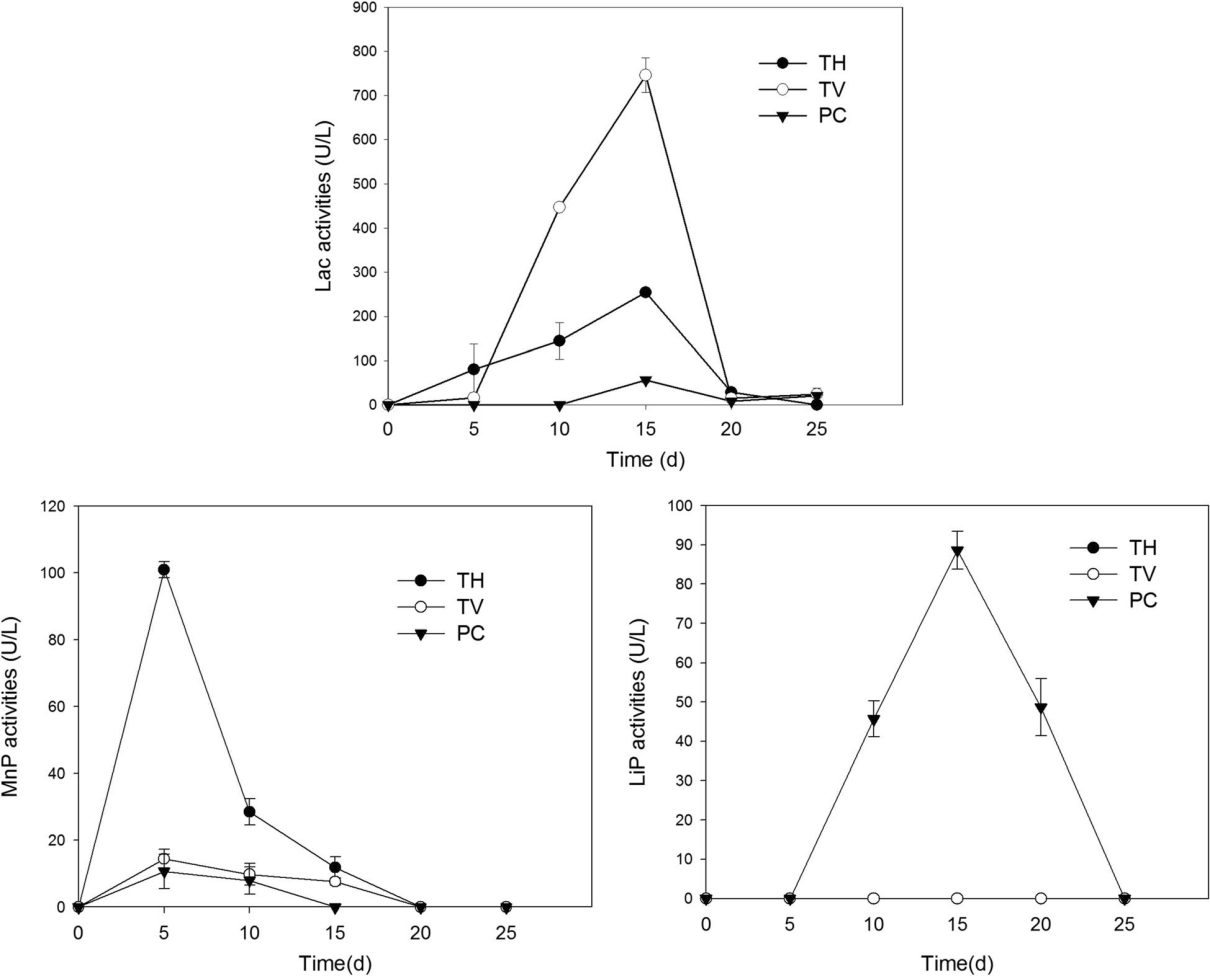
Lignin peroxidase, laccase and manganase peroxidase activities of *Phanerochaete chrysosporium*, *Trametes versicolor* and *Trametes hirsute* on tobacco stalk during solid fermentation. The culture temperature was 28 °C and the relative humidity was 80%, the enzyme activity was detected every 5 days until 25 days after inoculation

The highest activity of MnP (100.95 U · L^−1^) was observed in *T. hirsute* at an early stage after inoculation (Fig. 2). This result further proved that MnP is a lignin attack enzyme produced by basidiomycetes in the initial stage of lignin degradation [20]. On the other hand, a relatively higher Lac activity (254.72 U · L^−1^) was also observed with *T. hirsute*. Lip was found play an important role during the degradation of tobacco stalk for *P. chrysosporium* and the highest LiP activity (88.62 U · L^−1^) was achieved at 15 d after inoculation (Fig. 2). The highest activity of Lac (745.65 U · L^−1^) was achieved at 15 d after inoculation with *T. versicolor* (Fig. 2).

All of these ligninolytic enzymes may be potentially applied in biobleaching, biopulping and the biodegradation of aromatic environmental pollutants [21]. And fermentation of lignocelluloses by fungi is becoming an economical way for the production of these ligninolytic enzymes. In this study, Lac activity detected in *T. versicolor* was at a higher level compared with Wang et al. report (633.3 U · L^−1^) [22] emphasizing the necessity to use tobacco stalk as substrate for the production of Lac.

#### Cellulolytic enzyme produced during the solid state fermentation of tobacco stalk

**Fig. 3:**
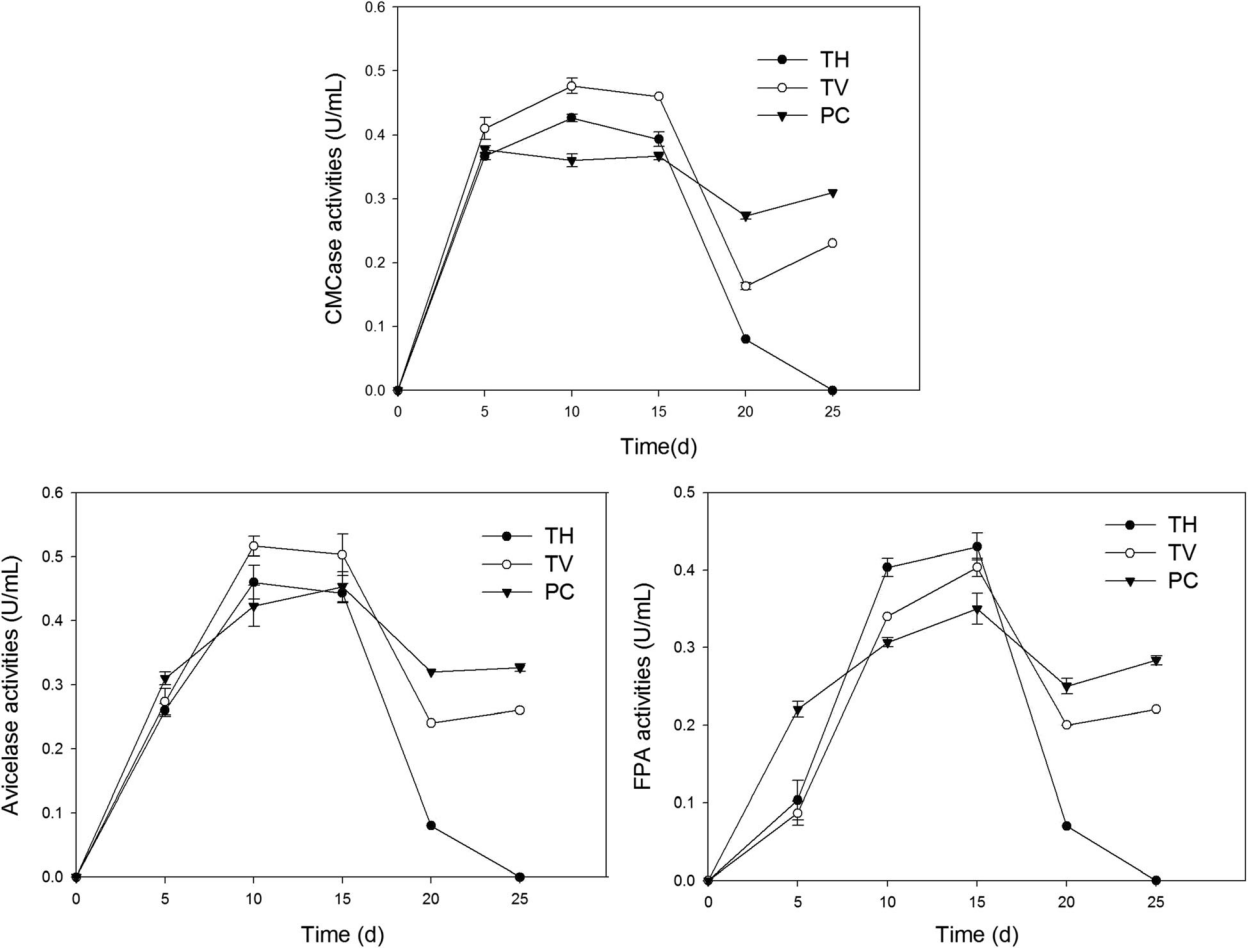
Production of cellulase by *Phanerochaete chrysosporium*, *Trametes versicolor* and *Trametes hirsute* when fermentation with tobacco stalk in solid state. The culture temperature was 28 °C and the relative humidity was 80%, the enzyme activity was detected every 5 days until 25 days after inoculation

Endoglucanase (E.C.3.2.1.4) (CMCase), exoglucanase (E.C.3.2.1.91) (avicelase) and filter paper enzyme (FPA) are the main cellulase produced in the degradation of cellulose. It was observed that all the fungi tested could secret a considerable amount of cellulolytic enzymes (Fig. 3).

The highest CMCase (0.51 U/mL) and avicelase activity (0.51 U/mL) were observed in *T. versicolor* and the highest FPA activity was observed in *T. hirsute* (0.43 U/mL). Though the highest cellulase activities were detected in *T. hirsute* or *T. versicolor*, their enzyme activities were unstable compared with *P. chrysosporium*. Cellulolytic enzyme activities in *T. hirsute* and *T. versicolo*r were sharply decreased at 20 days after inoculation and were significantly lower than *P. chrysosporium* (Fig. 3). Overall, *P. chrysosporium* could produce cellulase more consecutively, suggesting that it is more suitable for the production of cellulase by fermentation with tobacco stalk.

It can also conclude that the cellulolytic enzymes production of fungi strains was influenced by substrate. FPA was demonstrated to be the dominant cellulase produced by *T. versicolor* when fermentation with sorghum husks [23] and the highest CMCase activity was observed in *T. versicolor* when fermentation with wheat stalk [24]. However, almost the same amount of CMCase (0.48 U/mL) and avicelase (0.51U/mL) was produced by *T. versicolor* when fermentation with tobacco stalk (Fig. 3). *T. hirsute* showed a relatively higher CMCase enzyme activity when fermentation with paddy straw [25]. While the relatively higher FPA activity was observed in *T. hirsute* when using tobacco stalk as substrate (Fig. 3).

The enzymes secreted from the strains undergo a sharp decrease in activity in the late stages of the experiments. When a fungus was inoculated to the tobacco stalk, various enzymes were secreted to degradate the tobacco stalk. Even in the degradation of lignin, laccase, Lip and Mnp are the representative enzymes but not the whole enzymes in the degradation process. Furthermore, different enzymes might play different roles in different process. Therefore, to consider the production of enzymes using tobacco stalk in our subsequently research, the characteristic of the enzymes would be investigated including the stability and the inhibitor of the enzymes etc.

#### Relationship between enzyme producing and lignocelluloses degradation

It could be concluded that specific ligninolytic enzymes were produced by different fungi during the fermentation of tobacco stalk and lead to the different degradation efficiencies of lignin. LiPs are able to oxide the lignin compounds directly via long-range electron transfer and was considered as key enzymes in the degradation of lignin especially for *P. chrysosporium* [26]. The highest lignin degradation ability of *P. chrysosporium* is in accordance with the highest LiP activity detected in *P. chrysosporium*. The large molecular weight of Lac makes it difficult to penetrate into the lignocelluloses, and Lac was reported to play an important role in the initial stage of lignocellulose degradation [27]. That’s the reason why *T. versicolor* showed highest Lac activity but had lower lignin degradation efficiency.

## Conclusion

The nicotine in tobacco stalk could be degraded to a safe level after 15 days of fermentation with the three white rot fungi *Phanerochaete chrysosporium*, *Trametes versicolor* and *Trametes hirsute. Phanerochaete chrysosporium* is suitable for the degradation of lignin in tobacco with 53.75% lignin reduction after 15 days fermentation. A considerable amount of laccase (745.65 U · L^−1^) was produced by *Trametes versicolor* and a relatively higher cellulase was secreted by *Phanerochaete chrysosporium* during the degradation of tobacco stalk. Furthermore, the products and intermediate in the degradation of tobacco stalk with *P. chrysosporium* were qualified. Enzyme activity detection results and the products had indicated a new approach for the reusing of tobacco stalk.

## Methods

### Tobacco stalk and strains

The tobacco stalk was obtained from the tobacco-planting area of Zhucheng Shandong province in China. The stalk was dried at 80 °C for 12 h, ground and sieved through a 40 mesh size sieve and then stored in valve bag for further treatment. Tobacco stalk was autoclaved at 121 °C for 20 min prior to inoculation with strains.

Four fungi *Phanerochaete chrysosporium*, *Trametes versicolor* [28], *Trametes hirsute* [29], *Aspergillus* sp. which were widely used in the biodegradation of other crop straw and other screened strains were used in this research. Their capacity to utilize the tobacco stalk as the sole carbon resource had been screened. Based on the preliminary results, *P. chrysosporium*, *T. versicolor*and *T. hirsute* were chosen for the further research of degradation of lignin and nicotine. The strains were provided by Chengsheng Zhang, a researcher belongs to Tobacco Research Institute of Chinese Academy of Agricultural Sciences, Key Laboratory of Tobacco Pest Monitoring Controlling & Integrated Management, State Tobacco Monopoly Bureau.

### Solid state fermentation of tobacco stalk with selected fungi

Solid state fermentation was carried to verify the tobacco stalk degradation efficiency of the strains selected. 10 g dried tobacco stalk was added into a 100 mL conical flask with 40 mL distilled water and autoclaved at 121 °C for 20 min. The fungal organisms were precultured on PDA (Potato Dextrose Agar) medium for 2 weeks, and two agar plugs (5 mm in diameter) of the overgrown plates were used to inoculate solid-state microcosms. The flasks were incubated at 28 °C for 25 d without light. All the fungi were treated identically and the conical flask with no inoculation was used as control.

### Determination of the degradation of tobacco stalk

The tobacco stalk is mainly composed of lignocellulose and nicotine. Meanwhile, lignocellulose is composed of lignin, cellulose and hemicellulose, among which the abundance of lignin is higher than most of the other biomass. The content of lignin, cellulose and hemicellulose were detected in this study to verify the tobacco stalk degradation efficiency.

#### Determination of nicotine in tobacco stalk

The content of nicotine in tobacco stalk was determined after incubation with an interval of 5 days by the method described by Wang et al. [30] The fermentation was carried out in quintuplicate, and every group was sampled on day 5, 10, 15, 20 and 25, respectively, to make sure that the whole microenvironment was not disturbed. The fermentation product was extracted with hydrochloric acid (0.5 mol/L) at a ratio of 0.05 g/25 mL, then mixed with active carbon (0.25 g) for 5 min and filtered through filter paper. The absorptions of the extraction were monitored by a UV–vis spectrophotometer (UV 2550) at given wavelengths (236, 259 and 282 nm). The content of nicotine in tobacco stalk was calculated by the following formula:

$$ \mathrm{Nicotine}\left(\%\right)=1.059\times \left[{\mathrm{A}}_{259}{\textstyle \hbox{-} }0.5\times \left({\mathrm{A}}_{236}+{\mathrm{A}}_{282}\right)\right]\times 100\times 100/\mathrm{W}\times \left(1{\textstyle \hbox{-}}\mathrm{Water}\%\right)\times 34.3\times 1000; $$

In the formula, 1.059 is the compensation coefficient; A259, A236 and A282 are the absorbance values of reaction system at 259, 236 and 282 nm; W is the weight of sample; 34.3 is the specific extinction coefficient of nicotine.

#### Determination of lignocelluloses in tobacco stalk

Every fungus was inoculated into four groups of conical flasks which were sampled at 9, 11, 13, 15 d after inoculation, respectively, each group was tripled. The treated tobacco stalk was extracted with 50 mL distilled water at 200 rpm at 28 °C for 2 h. The suspension was filtered through filter paper, and the solid residue was collected and dried at 50 °C for 2 days to a constant weight. The fermented dried residue was milled in order to obtain homogeneous samples. A modified quantitative analysis program [31] was used to determine the content of lignocelluloses in tobacco stalk.

First, 0.5 g dried residue was autoclaved with 50 mL neutral detergent at 105 °C for 1 h, then filtered through sand core crucible, the solid residue was washed by distilled water (50 mL) and acetone (50 mL) and weighed (W1) after dried at 80 °C for 12 h. Second, the residual in the first procedure was autoclaved with 50 mL hydrochloric acid (2 mol/L, 50 mL) at 105 °C for 1 h. After being cooled, the washing and drying step was repeated as described above and the solid residue was weighed (W2). Subsequently, the residual was hydrolyzed with 72% H_2_SO_4_ (45 mL) for 3 h, then 45 mL distilled water was added and keep at room temperature for 12 h. The mixture was filtered and the residues was washed and dried as mentioned in the first step, then the third weigh (W3) was got. At last, the dried residue was calcined in a muffle burner at 550 °C for 3 h; the ash product was weighed (W4) after being cooled in the vacuum dryer. The contents of hemicellulose, cellulose and lignin were calculated by the following formulas:

$$ \mathrm{Hemicellulose}\left(\%\right)=\left(\mathrm{W}1\hbox{-} \mathrm{W}2\right)/0.5 \times 100\% $$

$$ \mathrm{Cellulose}\left(\%\right)=\left(\mathrm{W}2\hbox{-} \mathrm{W}3\right)/0.5\times 100\% $$

$$ \mathrm{Lignin}\left(\%\right)=\left(\mathrm{W}3\hbox{-} \mathrm{W}4\right)/0.5\times 100\% $$

#### Determination of fermentation products with GC-MS

To further characterise the lignin and nicotine degradation ability of the chosen fungi, fermentation products of *P. chrysosporium* which was more efficiently in the degradation of tobacco stalk was determined. The cultures were extracted by water and ethyl acetate respectively at a ratio of 1 g/10 mL for 2 h with a rotation of 180 rpm/min. Then the mixture was centrifuged at 12, 000 rpm for 10 min and the supernatant was collected for GC-MS analysis.

Products characterization was carried out by capillary GC-MS using an Agilent 5975C system chromatograph. A HP-INNOWAX capillary column (30 m × 0.25 mm × 0.25 μm, Agilent, Palo Alto, CA, USA) was used, with helium as the carrier gas at a flow rate of 1 mL · min^−1^. The following oven temperature program was carried out: 50 °C for 1 min, increase of 20 °C/min to 120 °C, then programmed from 120 to 250 °C at 25 °C/min, where it was held for 5 min. The injector temperature was maintained at 250 °C; ion source temperature 230 °C; EI 70 eV; mass range 35–300 m/z. A suitable amount of sample was injected in split injection mode with a 20:1 split ratio. Peak identification was based on the relative retention time and total ion mass spectral comparison with the external standard.

### Assay of enzyme activities during the degradation of tobacco stalk

Enzyme was extracted by sodium acetate (10/1, v/w) at pH 6.5 and agitated on a rotary shaker at 180 rpm for 30 min. The extraction was filtered using Whatman filter paper, the filtrate was used for enzyme assay.

#### Assay of ligninolytic enzyme activities during the degradation of tobacco stalk

Lignin peroxidase (LiP) (EC 1.11.1.14) activity was assayed by determining the oxidation of veratryl alcohol using H_2_O_2_ [32]. The activity of manganese peroxidase (MnP) (E.C. 1.11.1.13) was determined by monitoring the oxidation of Mn^2+^ using H_2_O_2_ [33]. Laccase (Lac) (E.C.1.10.3.2) activity was assayed by examining the oxidation of 2′-azino-bis (3-ethylbenzthiazoline-6-sulfonic acid) (ABTS) at 420 nm [34]. One unit activity is defined as the amount of enzyme that transformed 1 μmol of substrate per min.

#### Assay of cellulolytic enzyme activities during the degradation of tobacco stalk

Endoglucanase (E.C.3.2.1.4) (CMCase), exoglucanase (E.C.3.2.1.91) (avicelase) and filter paper enzyme activities (FPA) were measured spectrophoto-metrically as described by Beukes and Pletschke [35] based upon the color reaction between the degradation products (glucose) and DNS (3,5-Dinitrosalicylic acid). In all cases, one unit of enzyme activity is defined as 1 μmol of glucose equivalents released per min per mL of filtrate under the given conditions.

## Additional file

Additional file 1: The original data about the lignocellulosic content and enzyme activities detection. (XLS 3055 kb)

## Abbreviations

CMCase: Endoglucanase
FPA: Filter paper enzyme
Lac: Laccase
LiP: Lignin peroxidase
MnP: Manganese peroxidase
PC: *P. chrysosporium*, *Phanerochaete chrysosporium*
TH: *T. hirsute*, *Trametes hirsute*
TV: *T. versicolor*, *Trametes versicolor*
